# Effect of Open-Ended Coaxial Probe-to-Tissue Contact Pressure on Dielectric Measurements

**DOI:** 10.3390/s20072060

**Published:** 2020-04-06

**Authors:** Gertjan Maenhout, Tomislav Markovic, Ilja Ocket, Bart Nauwelaers

**Affiliations:** 1 Division Telemic, Department of Electrical Engineering (ESAT), KU Leuven, Kasteelpark Arenberg 10, Box 2444, 3001 Leuven, Belgium; tomislav.markovic@esat.kuleuven.be (T.M.); bart.nauwelaers@esat.kuleuven.be (B.N.); 2 imec, Kapeldreef 75, 3001 Heverlee, Belgium; ilja.ocket@imec.be

**Keywords:** dielectric measurement, contact pressure, biological tissues, measurement metadata, open-ended coaxial probe

## Abstract

Open-ended coaxial probes are widely used to gather dielectric properties of biological tissues. Due to the lack of an agreed data acquisition protocol, several environmental conditions can cause inaccuracies when comparing dielectric data. In this work, the effect of a different measurement probe-to-tissue contact pressure was monitored in the frequency range from 0.5 to 20 GHz. Therefore, we constructed a controlled lifting platform with an integrated pressure sensor to exert a constant pressure on the tissue sample during the dielectric measurement. In the pressure range from 7.74 kPa to 77.4 kPa, we observed a linear correlation of −0.31±0.09% and −0.32±0.14% per kPa for, respectively, the relative real and imaginary complex permittivity. These values are statistically significant compared with the reported measurement uncertainty. Following the literature in different biology-related disciplines regarding pressure-induced variability in measurements, we hypothesize that these changes originate from squeezing out the interstitial and extracellular fluid. This process locally increases the concentration of membranes, cellular organelles, and proteins in the sensed volume. Finally, we suggest moving towards a standardized probe-to-tissue contact pressure, since the literature has already demonstrated that reprobing at the same pressure can produce repeatable data within a 1% uncertainty interval.

## 1. Introduction

One of the first, large-scale literature surveys of dielectric properties of biological tissues was conducted in 1996 [1]. It presented the dielectric properties of 13 different biological tissues in a frequency range from 10 kHz to 100 GHz. Since 2010, the Foundation for Research on Information Technologies in Society (IT’IS foundation) started to collect all tissue-specific properties in an open-access database [2]. They offer files with dielectric properties for specific tissue types, where each of these files is an average dielectric model based on submitted literature data. This can improve the theoretical models and the design of sensors and/or applicators in different dielectric-based medical applications, e.g., dielectric imaging [3,4], hyperthermia [5,6], and ablation [7,8]. However, inaccuracies or errors in the submitted literature data can directly impact the sensitivity and effectiveness of these applications [9].

Errors can corrupt dielectric measurement data when the measured volume is too small [10], when the calibration is inaccurate [11] or when the heterogeneity of the sample is not taken into account [12]. Apart from these measurement errors, data can also be different, due to different temperatures [13], different hydration levels [14], or even a difference in the age of the tested sample source [15]. In this case, averaging the data could lead to a large deviation, due to a heterogeneous sample group. The field of dielectric measurements is not the only one that faces this challenge. However, other biology-related disciplines face the same challenge and propose the use of a standard framework. When measured data is reported, it must be accompanied with a minimum information model containing the relevant metadata. For instance, minimum information models are currently used in neuroscience (MINI model) [16], genomic investigations (MIBBI model) [17], and field spectroscopy [18].

In 2017, a similar framework (MINDER) was proposed for the metadata collection of dielectric measurements to obtain repeatable and reusable data [19]. This work identified all possible confounders that could influence the measurement of dielectric properties, e.g., the source of the tissue, calibration procedure, and environmental conditions. Currently, the total number of possible confounders makes this list unfeasible. Therefore, the influence of possible confounders must be examined and analyzed to verify whether or not their contribution is significant or not and whether or not they should be included in the minimum information model for conducting dielectric measurements.

Dielectric properties of tissues can be extracted with several techniques, e.g., the reconversion of measured scattering parameters of transmission lines [20] or a cavity perturbation [21]. During recent years, the slim form, open-ended coaxial probe has become the most applicable technique for dielectric measurements [22,23]. The probe’s position is fixed throughout the experiment to eliminate the introduction of inaccuracies. The tissue sample is placed on a movable platform. Next, an operator gently raises the sample until it contacts the probe. Unfortunately, there is no guideline on when the operator should stop, i.e., which probe-to-tissue contact pressure is desired and how it varies over time. The literature reveals that the probe-to-tissue contact pressure in other biology-related disciplines has a significant impact on the measurements of, for instance, pressure waves in acoustic radiation force (ARF)-based ultrasound elasticity measurements [24], low-frequency electromagnetic waves for electrical bioimpedance measurements [25,26,27], and electromagnetic waves in the visible light and infrared spectrum for measurements of optical properties [28]. These results suggest that the probe-to-tissue contact pressure is significant and cannot be neglected. To the authors’ best knowledge, no reports are currently available on the effect of this contact pressure regarding dielectric measurements of biological tissue and whether or not it is significant as well.

In this work, the effect of different probe-to-tissue contact pressure is investigated. Section 2 elaborates more on the mechanical tissue deformation during probing, how the measurement setup is constructed, as well as how measurements are conducted and analyzed. Section 3 presents the obtained results of seven conducted experiments. Here, an average experiment with 10 different measurements is also presented in more detail. Next, Section 4 discusses the obtained results and proposes a hypothesis to explain the results and link it with hypotheses from other biology-related domains. Finally, the main results are summarized.

## 2. Materials and Methods

Experiments were conducted on fresh bovine liver that was purchased at a local grocery shop. All samples were sliced from a single liver to limit the influence of other confounders, such as age [15], throughout all conducted experiments. The samples were hermetically sealed and stored in a fridge at 4 ${}^{\circ }$C before the experiments to prevent tissue degradation, due to putrefaction. Prior to the start of each individual experiment, a tissue sample of approximately 2 cm × 2 cm × 2 cm was taken out of the fridge, wrapped in aluminum foil to limit the effect of dehydration, and given time to acclimatize to room temperature, i.e., passive heat acclimation. A type T thermocouple was inserted to monitor the temperature of the sample. The acclimation step ended when the measured temperature remained constant during 2 min. Throughout all experiments, the average temperature of the samples was 18.2 ± 1.0 ${}^{\circ }$C. At the beginning of each experiment, the sample was unwrapped and dried with a paper towel to prevent the build-up of excess moisture on the tissue surface at the probe tip. Every experiment lasted approximately 30 min. During this time span, the sample was exposed to the open air conditions in our lab (ambient temperature of 19 ${}^{\circ }$C and relative humidity of 60%, measured with HDC1080EVM, Texas Instruments, Dallas, TX, USA). Our previous work [14] demonstrated that in these laboratory conditions at room temperature and for this duration, the effect of dehydration on the dielectric properties of the sample is not significant.

**Figure 1:**
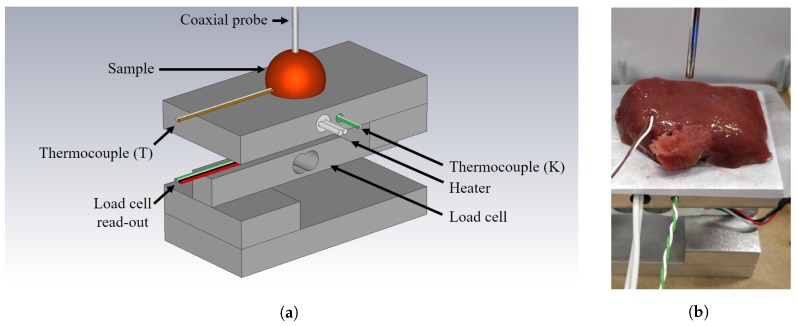
(**a**) Schematic overview of the used measurement setup with labeled components (note: the heater and thermocouple type K are not used in this work). (**b**) Photograph of the used measurement setup when a sample is loaded before the actual measurement.

**Figure 2:**
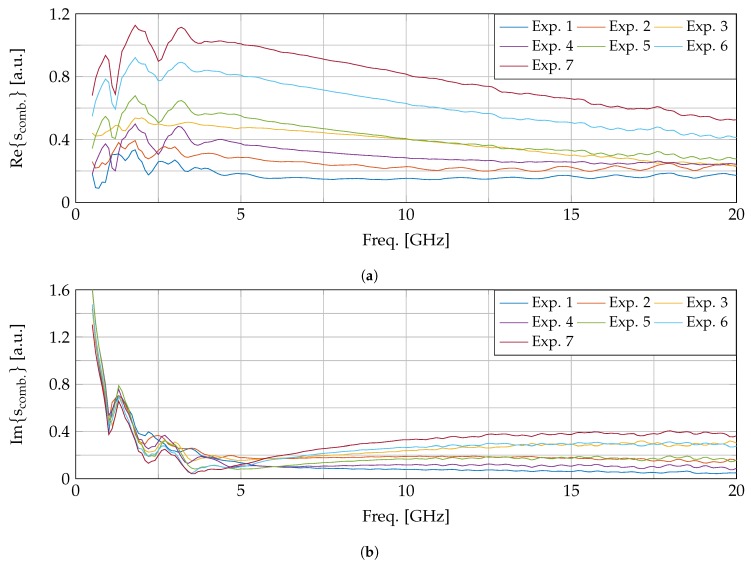
The calculated combined complex uncertainty s_comb_. for each conducted experiment, the real and imaginary part, in, respectively, (**a**,**b**).

The dielectric measurements were taken with a slim form, open-ended coaxial probe (N1501A, Keysight, Santa Rosa, CA, USA) connected with a Phaseflex 3GW40 cable (Gore, Newark, DE, USA) to our PNA (E8361, Keysight, Santa Rosa, CA, USA). The setup is displayed in Figure 1. The measurements were conducted in the 0.5 GHz to 20 GHz frequency range with a 100 MHz frequency step. An intermediate (IF) bandwidth of 100 Hz and a power level of –5 dBm were used as recommended in the coaxial probe guidelines. The probe was calibrated using the corresponding Keysight Materials Measurement Suite with the probe in air, with the probe connected to the shorting block and with the probe submerged in deionized water. Before and after every single experiment, a 0.1 M sodium chloride validation liquid was measured to assess the combined uncertainty of the experiment [29]. This combined uncertainty $s_{\mathrm{comb}.}$ includes the random uncertainty, the systematic uncertainty, and the drift throughout the experiment and is calculated as explained in [14]. In this work, we conducted 7 different experiments. For each experiment, the uncertainty levels were calculated at every frequency point and the results are shown in Figure 2. The mean uncertainty value over the complete frequency range and over all experiments equals 0.42 and 0.23 for, respectively, the real and imaginary part. These values are comparable to other reported uncertainty assessments in a similar frequency range [30,31,32]. We can note that the uncertainty of the imaginary part at frequencies below 1 GHz is higher compared to the rest of the frequency range. When analyzing the three aforementioned uncertainty components, it can be deducted that the combined uncertainty is largely dominated by the systematic uncertainty. This could be explained due to the ionic polarization of the 0.1 M sodium chloride solution. The higher uncertainty below 1 GHz makes the data in that frequency range more prone to errors, due to the measurement uncertainty which complicates statistically significant conclusions, as will be discussed further on.

At the start of each individual experiment, the sample was placed on the measurement setup. A schematic overview of our setup is shown in Figure 1a and a photograph in Figure 1b. The measurement setup is built around a load cell (SEN0160, DFRobot, Shanghai, China) with four aluminum blocks, that measures mass. The measured mass *m* is transformed to a pressure *P* using the gravitational constant *g*, the area of the probe tip *A* and the masses from the sample $m_{\mathrm{sample}}$ itself, and the setup $m_{\mathrm{setup}}$:

(1) $$P=\frac{(m-m_{\mathrm{sample}}-m_{\mathrm{setup}})\times g}{A}.$$

We constructed a lifting platform with a linear actuator and driver unit that was able to raise and lower the load cell with the sample on top. The lifting platform is inspired by the lab jack principle, where one of the two bottom bars is directly connected to the linear actuator. A microcontroller (Arduino Uno, Arduino, Ivrea, Italy) read out the mass of the load cell, related to the applied pressure, and controlled the driver unit (L298N Dual H-bridge, STMicroelectronics, Geneva, Switzerland) of the lifting platform.

During a first experiment, we raised the platform until a pressure of 7.74 kPa (corresponding with a mass of 3 g) was reached. This position was maintained and we monitored an exponential decay in pressure P, with regards to the initial pressure $P_{\mathrm{init}.}$, the equilibrium pressure $P_{\mathrm{eq}.}$ and the time constant time constant $t_{c}$:

(2) $$P=P_{\mathrm{eq}.}+\left(P_{\mathrm{init}.}-P_{\mathrm{eq}.}\right)\times \exp\left(-\frac{t}{t_{c}}\right).$$

**Figure 3:**
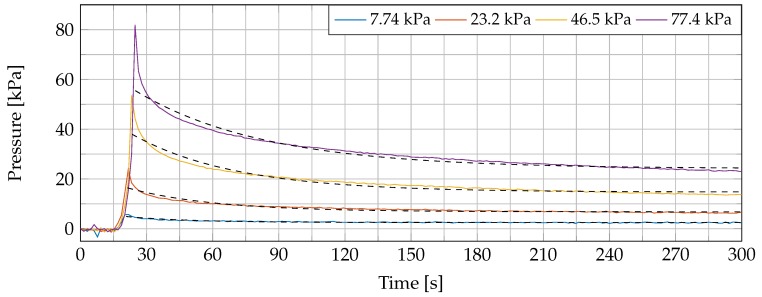
The exponential decaying behavior of the tissue: the tissue starts to conform according to the probe tip after applying an initial pressure. The decaying process is fitted with an exponential model and represented with dashed black curves.

We believe that the tissue conformed its shape to the probe, which resulted in a pressure redistribution over time. Next, the dielectric probe was released and a two-minute rest period was incorporated to let the sample recover to its initial state following the employed procedure in [26]. The measurement was repeated nine times at the same sample location at linearly, gradually increasing pressures from 15.5 kPa (6 g) to 77.4 kPa (30 g) while incorporating the same recovery interval between measurements. An exponential decay of applied probe-to-tissue contact pressure over time was monitored in each measurement. This effect is displayed in Figure 3 for four different pressure levels with a fitted exponential curve through these values (dashed black lines in the same figure). The values of the exponential fitting procedure are shown in Table 1 for the 10 different pressure levels. A similar exponential decay was reported in [33].

Due to the large time constant $t_{c}$ of the exponential decay and due to the large difference between the initial pressure $P_{\mathrm{init}.}$ and equilibrium pressure $P_{\mathrm{eq}.}$, we programmed a PI-controlled (proportional-integral controlled) pressure loop on the microprocessor to reach a more stable pressure, closer to the desired one, in a shorter amount of time. Seven different experiments were conducted. In each experiment, a fresh sample was placed on the measurement setup, it was raised until the PI-control reached the desired pressure, at which point the dielectric measurement was started. Following every single measurement in the experiment, the probe was released to let the sample recover, as previously explained. After this rest period, the next measurement was performed on the same location of the sample to limit any variation that could arise from tissue heterogeneity. Throughout every single experiment, 10 separate measurements at linearly, gradually increasing pressures were recorded (from 7.74 kPa to 77.4 kPa).

## 3. Results

**Figure 4:**
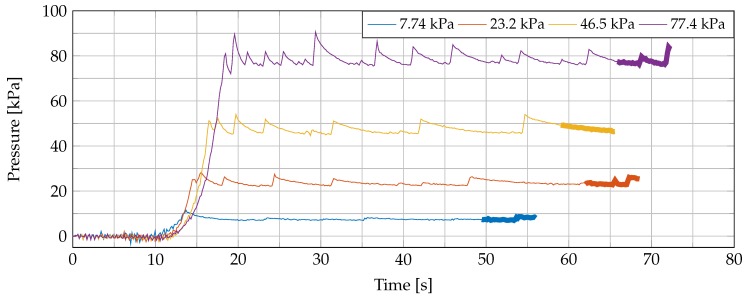
The measured pressure during four measurements of Experiment 5 with our PI-controlled pressure loop. The dielectric measurement is performed during the time span indicated as a thicker line.

**Figure 5:**
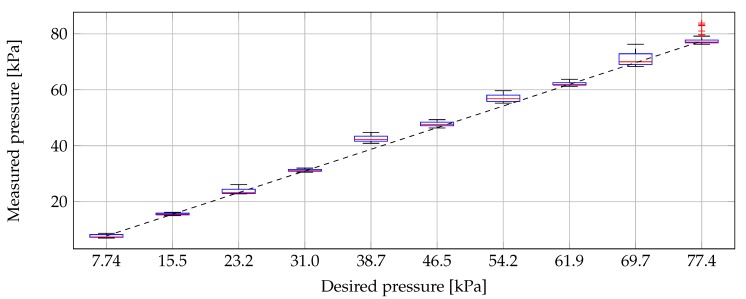
The actual measured pressure during the dielectric measurements of Experiment 5, graphically presented in a box plot, with respect to the desired pressure (dashed black line). (The horizontal red line indicates the median value and the blue box ranges from the 25th to the 75th percentile, respectively called Q${}_{1}$ and Q${}_{3}$. The minimum and maximum values are indicated with the horizontal black lines except if they are smaller than Q${}_{1}-\frac{3}{2}\times$ (Q${}_{3}$ – Q${}_{1}$) or larger than Q${}_{3}+\frac{3}{2}\times$ (Q${}_{3}$ – Q${}_{1}$). In that case, they are represented with a red +.).

With our PI-controlled pressure loop, we obtained a much stabler probe-to-tissue contact pressure in a shorter time compared to the initial contact-and-stop procedure. In this section, we present the obtained results of the seven conducted experiments and give more details about the measurements throughout a single experiment (Experiment 5 was chosen for this purpose because it represents an average case, as can be seen in the following figures). Four recorded pressures throughout Experiment 5 are shown in Figure 4. The dielectric measurement of the sample under test was conducted during the time span that is indicated with a thick line. During all 10 dielectric measurements of Experiment 5, the pressure was analyzed and represented in box plot format in Figure 5. There, we included a black dashed line as a reference for the desired pressures. We can observe that the mean pressure level at the dielectric measurement is close to the desired one and that the deviation of the applied pressure is limited.

**Figure 6:**
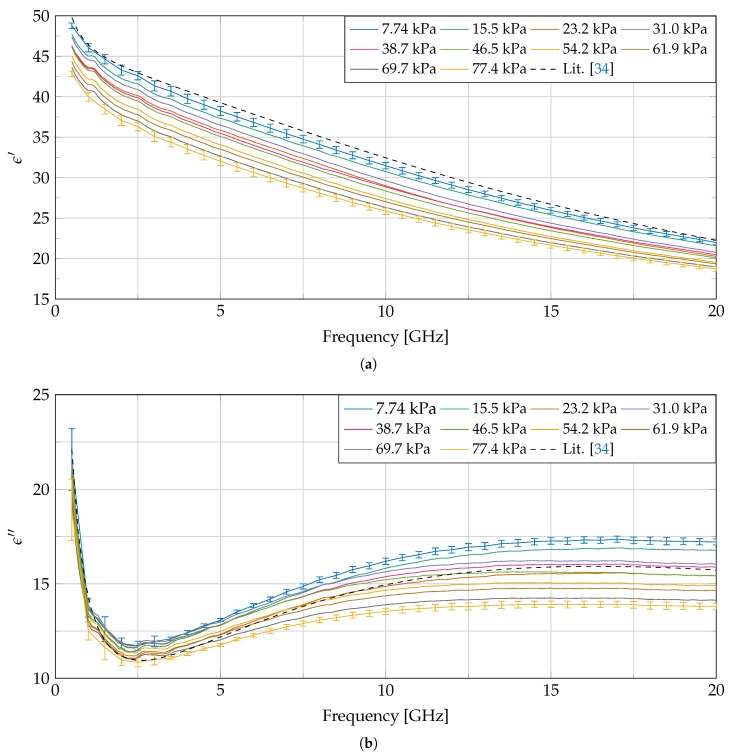
Measured dielectric properties (real and imaginary in respectively (**a**,**b**)) throughout Experiment 5 at 10 different pressures and compared with the dielectric properties for liver found in [34]. The error bars from this experiment are calculated as explained in Section 2 and are added to the measurement at the lowest and highest pressure.

Throughout every of the seven experiments, 10 dielectric measurements are conducted, each at a different linearly, gradually increased pressure level. For the case of Experiment 5, the measured complex permittivity is shown in Figure 6. Error bars, calculated as described in Section 2, indicate the extended uncertainty for the measurement at the lowest as well as the one at the highest applied pressure (other error bars were omitted to maintain figure readability). They demonstrate that a statistically significant difference in dielectric properties is present, due to a difference in applied pressure. All 10 measurements, apart from the one at 31.0 kPa, indicate a monotonic decrease in the real as well as imaginary part of the complex permittivity over the complete frequency range. However, we cannot claim that the loss of monotonicity at the measurement at 31.0 kPa is statistically significant, due to the aforementioned reported measurement uncertainty. The permittivity of liver from in vitro measurements at 37 ${}^{\circ }$C, found in the literature [34] which is used in the IT’IS database [2], is added as a reference. Due to the higher temperature of the sample used in [34], we would expect to see a lower value for $\epsilon^{\prime }$ and a higher value for $\epsilon^{\prime \prime }$. However, we observe an opposite trend, which could be attributed by difference in applied pressure, by tissue dehydration before the experiment [31] or by difference in dielectric properties between human and bovine liver.

**Figure 7:**
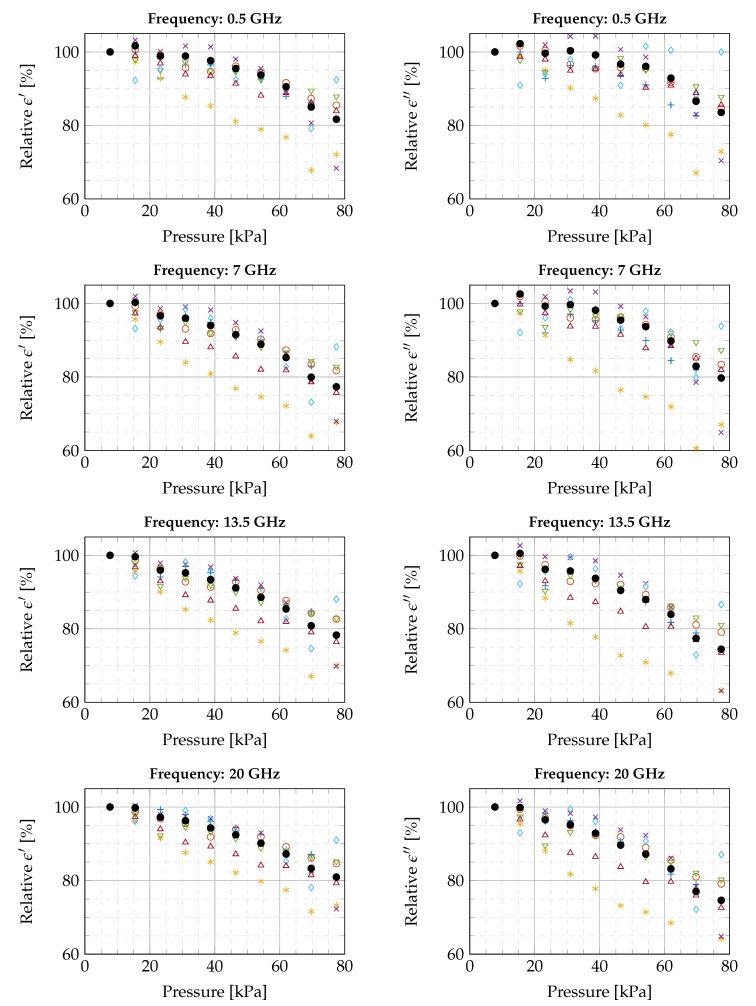
Overview of the relative changes of all conducted experiments as a function of pressure, presented at four linearly spaced frequencies in the measured frequency interval. (Legend: Experiment 1 +, Experiment 2 ○, Experiment 3 ✶, Experiment 4 ×, Experiment 5 ∇, Experiment 6 ✧, Experiment 7 ∆, and the average of all seven experiments ●

A similar decrease in dielectric properties over the complete frequency range is observed in the other six experiments. To compare all experiments with each other and to deduce trends in dielectric properties as a function of applied pressure, we normalized the measurements of every experiment to the measurement at the lowest pressure (7.74 kPa). The obtained relative, real and imaginary, dielectric properties are shown in Figure 7 for four linearly spaced frequencies over the measured frequency range. For every relative value at every frequency in each experiment, we observe a linear decreasing behavior in dielectric properties for increasing probe-to-tissue contact pressure. The values of these linear slopes, in the range from the lowest to the highest pressure value, are presented in Table 2 for four linearly spaced frequencies throughout the seven conducted experiments. The average relative change was calculated as well at every recorded frequency point throughout the seven conducted experiments. This yielded an average relative change of −0.31±0.09% and −0.32±0.14% in, respectively, the real and imaginary part of the complex permittivity per kPa.

Finally, we observe an increasing cross-experiment variability for increasing pressure. A possible explanation for this behavior could originate in the applied stress on the sample. With increasing pressure, the induced stress on the sample increased as well. It is possible that at the highest pressure the tissue was punctured and that interstitial fluid could have been released. The release of this interstitial fluid at the tip of the probe could result in an increase in measured permittivity. However, when visually inspecting the sample after the experiment, we did not notice this, but this could be because the amount of fluid was too small.

## 4. Discussion and Future Work

As demonstrated in Figure 6 and Figure 7, a statistically significant decrease in dielectric properties is observed. Since we are measuring every sample at the same position to minimize variation in sample heterogeneity, since the temperature of the sample is stable, and since dehydration at this temperature for this duration is insignificant, we believe that the observed difference in dielectric properties can only occur from the difference in applied pressure. We believe that applying different pressures changes the cellular matrix, which results in a different electromagnetic response. Moreover, the effect of pressure-dependent measurement responses is not limited to this specific domain, but similar trends are visible in other biology-related disciplines, as mentioned before [24,25,26,27,28].

With respect to electromagnetic responses at different pressure levels, bioimpedances have been measured from 9.6 kHz to 153.6 kHz by [26], where they noted an increase in resistivity. They hypothesize that the increase of pressure squeezes the intracellular fluid away and reduces the extracellular space, and that this is responsible for the increased resistivity measurements. For higher frequencies (100 Hz to 1 MHz), [35] noted an increase in resistivity and a decrease in capacitance for measurements with increasing pressure. They attribute these changes to intracellular fluid losses to the extracellular space throught the cell membrane, a reshaping of the cells, and contacting cell membranes that serialize cell membranes and decrease capacitance. In other disciplines, squeezing away intracellular and interstitial fluid is routinely used to explain pressure-induced changes in biological properties [28,33].

The measurements are conducted in the frequency range from 500 MHz to 20 GHz. This means that we are above the α- and β-dispersion and that the measurements should not dependent on the shape of the cell nor on the dipolar moments related to the cell membrane [36]. Therefore, we can roughly approximate the tissue as a two-phase composite with two separate volume fractions. The first phase represents several fluids, e.g., cytoplasm and interstitial fluids, which can be approximated as a saline solution. The second phase contains all other inclusions, e.g., bipolar lipid membranes, cell organelles, and proteins. If we would continue the same line of reasoning as in the aforementioned literature, i.e., pushing away interstitial and intracellular fluid, then the sensed volume would contain a higher concentration of proteins and membranes when applying pressure. Both of them typically have a lower complex permittivity value than a saline solution, as shown in [37,38], respectively. Given the composition of the liver [39], the cytoplasmic matrix and the intercellular spaces (host medium) are responsible for $\varphi_{\mathrm{host}}=64.73\%$ of the liver volume. When we assume that this host volume is filled with a 0.1 M saline solution with permittivity $\epsilon_{\mathrm{host}}$, we can calculate the complex permittivity of the inclusions $\epsilon_{\mathrm{incl}.}$ from the effective permittivity $\epsilon_{\mathrm{eff}.}$ obtained during the lowest pressure measurement, using the Landau–Lifshitz–Looyenga mixing formula [40]:

(3) $$\epsilon_{\mathrm{eff}.}={\left(\varphi_{\mathrm{host}}\times \sqrt[{3}]{\epsilon_{\mathrm{host}}}+\left(1-\varphi_{\mathrm{host}}\right)\times \sqrt[{3}]{\epsilon_{\mathrm{incl}.}}\;\right)}^{3}$$

**Figure 8:**
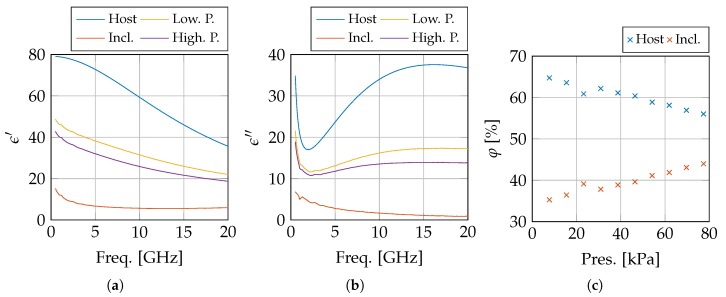
The dielectric properties of the host medium, the inclusions, and the compared to the measurements at 7.74 kPa (Low. P.) and 77.4 kPa (High. P.) with the real and imaginary part in, respectively, (**a**,**b**). The calculated volume fractions j are displayed in (**c**).

Using $\epsilon_{\mathrm{host}}$ and the extracted $\epsilon_{\mathrm{incl}.}$, we can calculate the volume of the host medium $\varphi_{\mathrm{host}}$, i.e., the amount of fluid in the sensed volume, throughout the measurements at different pressure levels by treating each of them as a new $\epsilon_{\mathrm{eff}.}$. This demonstrates as shown in Figure 8, assuming that the inclusions do not move, a decrease in the volume fraction of the host medium from 64.73% to 56.01% for an applied pressure of 7.74 kPa and 77.4 kPa, respectively.

Since the effect of pressure on dielectric measurements is significant (average relative change of −0.31±0.09% and −0.32±0.14% per kPa), we suggest that a constant, standardized pressure should be exerted throughout the duration of the measurement. Since it is difficult to optically verify the applied pressure and since probe displacement in itself does not contain sufficient information to characterize the applied pressure [26], we believe that the probe-to-tissue contact pressure should be reported in the metadata list for each dielectric measurement. Secondly, we observed an exponential decaying applied pressure with a large time constant when using a straightforward contact-and-stop procedure. When data is gathered during this decaying time period, the pressure can differ throughout the measurement which could result in inaccuracies in the acquired data. Thirdly, a standardized pressure should be proposed in an agreed data acquisition protocol that can be achieved in ex vivo measurements and that is relevant for in vivo measurements as well where there is no solid surface backing the sample during the measurement. With such a standardized probe-to-tissue contact pressure, it has been demonstrated in [41] that reprobing can deliver accurate dielectric measurements with an uncertainty of 1% in the 800 to 2450 MHz range.

## 5. Conclusions

In this work, we evaluated the effect of applied probe-to-tissue contact pressure on the measured dielectric properties. The tissue under test was bovine liver at a temperature of 18.2 ${}^{\circ }$C. First, we evaluated the mechanical effect of probing the tissue. Here, we noted an exponential decaying pressure with time constants up to 58 seconds. Since we were interested in a constant exerted pressure of the probe on the tissue, we implemented a PI-controlled lifting platform that allowed conducting dielectric measurements under constant probe-to-tissue contact pressure.

At 10 different pressure levels from 7.74 kPa to 77.4 kPa, we measured the dielectric properties of seven different bovine liver samples. For each sample, we observed a statistically significant difference in dielectric properties when measuring with a different applied pressure. The average observed relative changes in the real and imaginary part of the complex permittivity are −0.31±0.09% and −0.32±0.14% per kPa, respectively. We hypothesize that these changes originate from squeezing out the interstitial and intercellular fluid and, therefore, increasing the concentration of membrane, proteins, and cell organelles in the sensed volume. We strongly suggest that the exerted pressure should be stable during dielectric measurements and that a standardized pressure should be proposed in an agreed data acquisition protocol for dielectric measurements, as already exists in other biology-related disciplines.

## Figures and Tables

**Table 1:** Fitted exponential parameters from the pressure-induced exponential decay as shown in Figure 3 with the corresponding goodness of fit value ($R^{2}$).

Desired Pressure [kPa]	$P_{\mathrm{init}.}$ [kPa]	$P_{\mathrm{eq}.}$ [kPa]	$t_{c}$ [s]	$R^{2}$
7.74	7.37	2.50	29.33	0.85
15.48	13.93	4.07	35.32	0.89
23.23	23.29	6.78	39.65	0.91
30.97	24.98	8.65	50.14	0.93
38.71	36.44	11.78	50.88	0.94
46.45	52.99	14.72	47.01	0.94
54.19	50.28	16.37	55.34	0.95
61.94	63.21	19.41	53.85	0.95
69.68	64.45	21.71	57.43	0.95
77.42	72.08	24.13	58.63	0.96

**Table 2:** Linear slopes of relative changes in complex permittivity per kPa at the frequencies from Figure 7.

Meas.	$\Delta \left(\mathrm{Relative}\;\epsilon^{\prime }\right)/\mathrm{Pressure}[ \frac{\%}{\mathrm{kPa}}]$				$\Delta \left(\mathrm{Relative}\;\epsilon^{\prime \prime }\right)/\mathrm{Pressure}\left[\frac{\%}{\mathrm{kPa}}\right]$			
***Freq. [GHz]***	***0.5***	***7.0***	***13.5***	***20.0***	***0.5***	***7.0***	***13.5***	***20.0***
Exp. 1	−0.21	−0.28	−0.25	−0.25	−0.27	−0.29	−0.35	−0.40
Exp. 2	−0.21	−0.26	−0.24	−0.21	−0.23	−0.25	−0.30	−0.31
Exp. 3	−0.45	−0.50	−0.46	−0.40	−0.46	−0.54	−0.58	−0.56
Exp. 4	−0.41	−0.42	−0.38	−0.34	−0.38	−0.46	−0.47	−0.44
Exp. 5	−0.16	−0.24	−0.23	−0.20	−0.15	−0.15	−0.25	−0.26
Exp. 6	−0.16	−0.27	−0.27	−0.23	0.02	−0.14	−0.27	−0.28
Exp. 7	−0.23	−0.34	−0.32	−0.29	−0.20	−0.26	−0.37	−0.38

## Abbreviations

The following abbreviations are used in this manuscript:

ARF	Acoustic Radiation Force
IF	Intermediate Frequency
IT’IS	Foundation for Research on Information Technologies in Society
MIBBI	Minimum Information for Biological and Biomedical Investigations
MINDER	Minimum information for dielectric measurements of biological tissues
MINI	Minimum Information about a Neuroscience Investigation
PI-controller	Proportional-Integral-controller
PNA	Performance Network Analyzer
